# Dichotomous STAT5 and STAT6 Activation in T Cells Reflects Cytokine Shifts Between Blood and Skin in Atopic Dermatitis

**DOI:** 10.1111/all.16492

**Published:** 2025-01-30

**Authors:** Andreas Boldt, Regina Treudler, Marie Rabe, Niklas Artz, Sabine Seiffert, Rachael Bogle, Xianying Xing, Louise Wilkens, Ariane Bialas, Lea Präger, Lam C. Tsoi, Ulrike Koehl, Sandra Franz, Sonja Grunewald, Jan C. Simon, J. Michelle Kahlenberg, Johann E. Gudjonsson, Benjamin Klein

**Affiliations:** ^1^ Institute of Clinical Immunology University of Leipzig Leipzig Germany; ^2^ Department of Dermatology, Allergology and Venereology Leipzig University Medical Center, University of Leipzig Leipzig Germany; ^3^ Institute of Allergology Charité Universitätsmedizin Berlin Berlin Germany; ^4^ Department of Dermatology University of Michigan Ann Arbor Michigan USA; ^5^ Department of Biostatistics University of Michigan Ann Arbor Michigan USA; ^6^ Department of Computational Medicine and Bioinformatics University of Michigan Ann Arbor Michigan USA; ^7^ Department of Internal Medicine, Division of Rheumatology University of Michigan Ann Arbor Michigan USA

To the Editor,

Atopic dermatitis (AD) is a chronic inflammatory skin disease that can be targeted through inhibition of the Janus kinases (JAK) and Signal Transducers and Activators of Transcription (STAT) pathway [1, 2]. In the serum of AD patients, numerous cytokines activating this pathway are elevated. In the skin, IL‐13 contributes to inflammation through STAT6 [1, 3]. Moreover, gain‐of‐function mutations in STAT5 and STAT6 underly hereditary atopic disorders [4]. Enhanced T cell activation in AD has been observed, but it is currently unclear whether T cells in AD exhibit STAT signatures. Here, we compared STAT activation in AD CD4^+^ and CD8^+^ T cells in blood and skin. Adult AD patients (AD, *n* = 22) and healthy controls (*n* = 20) were recruited for this study (Data S1). Disease severity was assessed by the Eczema Area and Severity Index (EASI). We previously established measurements of pSTATs by flow cytometry and assessed pSTAT at baseline and after cytokine stimulation (Figures 1A–D, S1A).

**FIGURE 1:**
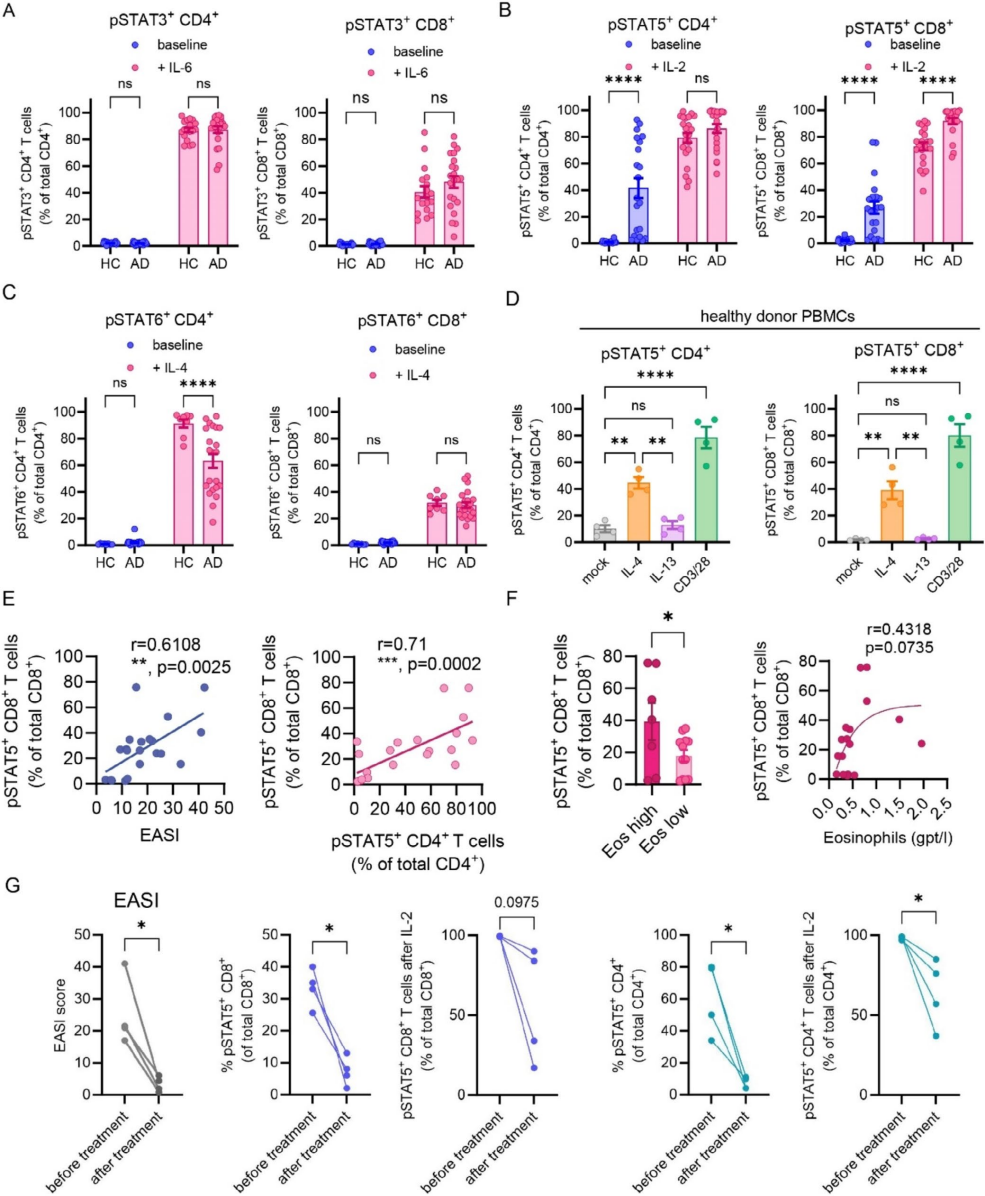
STAT5 activation in peripheral T cells in AD corresponds to disease activity. (A–D) Frequencies of T cells positive for pSTAT3 (A), pSTAT5 (B, D), pSTAT6 (C) in HC and AD at baseline and after cytokine stimulation. HC PBMCs were stimulated with IL‐4, IL‐13, or CD3/28, respectively (D). (E) Correlation of pSTAT5^+^ CD8^+^ T cells with the Eczema Activity and Severity Index (EASI) and with pSTAT5^+^ CD4^+^ T cells. (F) Comparison of pSTAT5^+^ T cells in patients with peripheral eosinophilia (> 0.5gpt/l, Eos high) to patients without eosinophilia (< 0.5gpt/l, Eos low) and correlation of pSTAT5^+^ T cells with eosinophils. (G) EASI and pSTAT5^+^ T cells before and after treatment in 4 patients. Two way ANOVA followed by Sidak's multiple comparisons test (A‐D). Linear Regression (E). Students unpaired (F) or paired (G) t‐test. Pearson correlation coefficient (E). Mean and SEM. *p < 0.05, **p < 0.01, ****p < 0.0001.

pSTAT3 levels were similar between HC and AD at baseline and after stimulation (Figure 1A). In contrast to STAT3, T cells exhibited a dramatic increase in the percentage of pSTAT5^+^ cells at baseline in AD compared to HC in CD4^+^ and CD8^+^ T cells (Figures 1B, S1B). Surprisingly, pSTAT6 revealed no differences in AD T cells versus HC (Figure 1C). Given the strong pSTAT5 phenotype, we stimulated PBMCs with IL‐4 and IL‐13, revealing an increase after IL‐4 but not IL‐13 (Figure 1D), indicating common gamma chain cytokines (γc) upstream of STAT5.

Importantly, the frequency of pSTAT5^+^ T cells correlated well with disease activity (Figures 1E, S1C), and higher pSTAT5 levels were observed in patients with peripheral eosinophilia (Figure 1F). We saw no correlation of pSTAT5^+^ T cells with serum IgE or age and no difference in individuals with atopic comorbidities, IgE‐mediated sensitizations, or intrinsic/extrinsic AD. Patients with hymenoptera venom allergy exhibited similar pSTAT5^+^ cells as HC (Figure S1). To determine if treatment impacts pSTAT5^+^ T cell frequency, we measured pSTAT5^+^ T cells in AD patients treated with either monoclonal antibodies against IL‐13/IL4Rα or JAK inhibitors and one patient who received systemic steroids (Data S1). Importantly, EASI response was accompanied by reduction of peripheral pSTAT5^+^ T cells (Figures 1G, S1G).

As T cells drive skin inflammation in AD, we performed immunohistochemistry that revealed minimal pSTAT5^+^ cells in lesional AD skin but strong pSTAT6 staining (Figures 2A, S2A). To further dissect which subsets are enriched in STAT5 activation in lesional AD skin, we applied a STAT5 signature to a scRNA sequencing dataset from lesional AD skin [5]. Surprisingly, skin T cells did not exhibit a STAT5 signature except for naïve CD8^+^ T cells (Figures 2B, S2B). We hypothesized that the cutaneous transcriptional signature might be changed due to a different cytokine microenvironment. Hence, a STAT6 signature was applied to skin T cell subsets, showing that T cells harbor a robust STAT6 response in AD skin compared to HC (Figure 2C). Interestingly, naïve T cells exhibited no STAT6 signature (Figure S2C).

**FIGURE 2:**
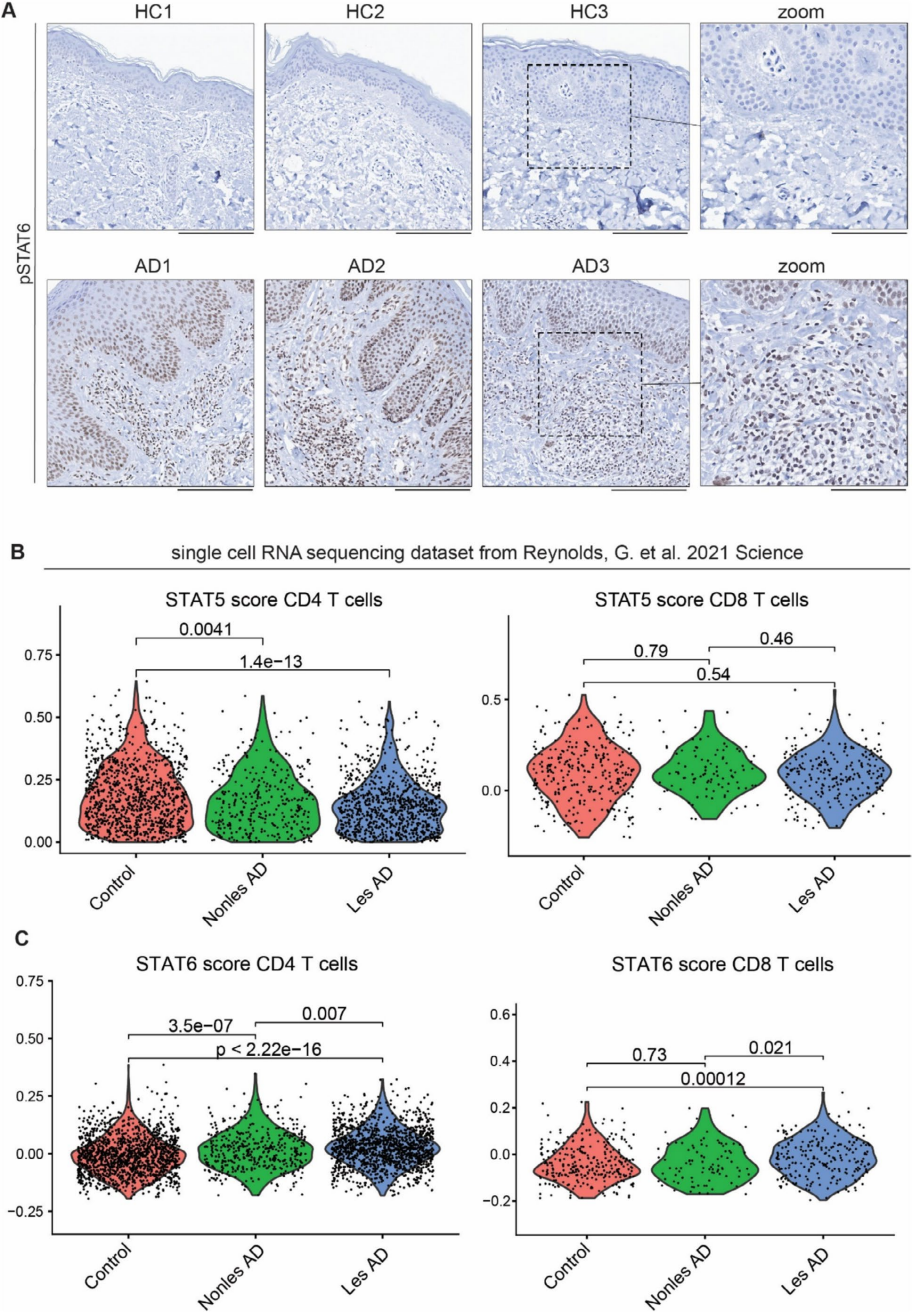
Cutaneous T cells exhibit a strong STAT6 signature in AD lesional skin. (A) Representative IHC images for pSTAT6 in HC (*n* = 3) versus lesional AD skin (*n* = 3), scale bar 200μm, in zoomed pictures 60μm. (B, C) Violin plots for STAT5 (B) or STAT6 (C) positive scores in different T cell subsets in healthy (Control), nonlesional AD (Nonles AD) and lesional AD (Les AD) skin from a previous scRNA sequencing dataset [5].

Here, we identify dichotomous STAT activation in AD, with circulating pSTAT5^+^ T cells reflective of γc signaling, corresponding to disease activity. STAT5 is required for the survival, proliferation, activation, and differentiation of Th2 cells [6]. As up to 80% of T cells were positive for pSTAT5, we suppose that this population represents T cells that will undergo differentiation. In the skin, most T cell subsets were positive for a STAT6 signature, indicative of a cytokine switch towards other cytokines such as TSLP and IL‐13. Thus, these data hint at a dichotomy in AD pathogenesis, with a separation between signals that act centrally, reflecting overall disease severity, and those in the skin proximal to the site of skin inflammation. Further studies, including proteomics and analysis of different AD endotypes, are needed to ultimately understand cytokine signaling in AD.

## Author Contributions

Conceptualization: Benjamin Klein, Johann E. Gudjonsson. Methodology: Andreas Boldt, Sabine Seiffert, Sonja Grunewald, Rachael Bogle, Johann E. Gudjonsson, Benjamin Klein. Investigation and data acquisition: Benjamin Klein, Ariane Bialas, Regina Treudler, Marie Rabe, Niklas Artz, Louise Wilkens, Ariane Bialas, Rachael Bogle, Lea Präger, Ariane Bialas, Johann E. Gudjonsson, Xianying Xing. Funding acquisition: Benjamin Klein. Visualization: Benjamin Klein, Ariane Bialas, Sabine Seiffert, Rachael Bogle. Project administration: Benjamin Klein, Niklas Artz, Johann E. Gudjonsson. Supervision: Benjamin Klein, Regina Treudler, J. Michelle Kahlenberg, Johann E. Gudjonsson. Writing – original draft: Benjamin Klein, Ariane Bialas. Writing – review and editing: Benjamin Klein, Ariane Bialas, Regina Treudler, Marie Rabe, Ulrike Koehl, Lam C. Tsoi, Sandra Franz, Jan C. Simon, J. Michelle Kahlenberg, Johann E. Gudjonsson.

## Conflicts of Interest

B.K., A.B., M.R., S.S., L.W., X.X., Ar.B., L.P., and S.F. declare no conflicts of interest. N.A. reports personal fees from Sun Pharma. R.T. reports grants and personal fees from Sanofi‐Genzyme, personal fees from ALK‐Abello, Takeda, Novartis, CSL Behring, AbbVie, and LeoPharma, and other from Fraunhofer‐IZI Leipzig, which are all independent of the submitted work. L.T. received support from Janssen and Novartis. U.K. received consultant and/or speaker fees from AstraZeneca, Affimed, Glycostem, GammaDelta, Zelluna, Miltenyi Biotec, Novartis, and Bristol‐Myers Squibb. S.G. received personal fees from Galderma, not related to this study. J.C.S. reports grants and personal fees from Sanofi‐Genzyme and Novartis, and personal fees from Lilly, Novartis, AbbVie, and LeoPharma. J.M.K. has received grant support from Q32 Bio, Celgene/Bristol‐Myers Squibb, Ventus Therapeutics, Rome Therapeutics, and Janssen. J.M.K. has served on advisory boards for AstraZeneca, Bristol‐Myers Squibb, Eli Lilly, EMD serrano, Gilead, GlaxoSmithKline, Aurinia Pharmaceuticals, Rome Therapeutics, and Ventus Therapeutics. J.E.G. has served on advisory boards for Bristol‐Myers Squibb, Eli Lilly, Janssen, Boehringer Ingelheim, UBC, Novartis, Almirall, Oruka Therapeutics, Takeda, and AbbVie.

## Supporting information


Data S1.

## Data Availability

The data that support the findings of this study are available from the corresponding author upon reasonable request.
