# Supplementary material for: Dichotomous STAT5 and STAT6 Activation in T Cells Reflects Cytokine Shifts Between Blood and Skin in Atopic Dermatitis
Source: Allergy. 2025 Jan 30;80(8):2379–83. doi: 10.1111/all.16492 (PMC12368756; doi:10.1111/all.16492)
Supplement: Supplementary file 1 — Data S1. [file ALL-80-2379-s001.docx]

**Supplemental Information**

**Methods**

**Patient characteristics and blood samples**

Adult AD patients (n=22, table 1), diagnosed according to criteria of Hanifin and Rajka^1^ without current systemic treatment and with active skin lesions were recruited through the Department of Dermatology in the comprehensive allergy center at Leipzig University Medical Center, Germany. Topical steroids were reportedly used in 67% of the patients, although we only included patients using low-moderate topical steroids (class VII-IV). We selected our patient cohort to include a wide variety of disease severity as measured by EASI score for correlation analysis. All subjects agreed to participate in an optional immunophenotyping assessment, approved by the local ethics committee (approval number 552/21-ek) after informed consent. We assessed disease activity by eczema area and severity index (EASI) to exclude subjective parameters such as itch and sleep loss^2^. In addition, we enrolled 20 age and gender matched HCs without known infection and five non-atopic patients with hymenoptera venom allergy (HVA) as disease controls for selected activation pathways.

**Table 1. Clinical characteristics of study participants**

| **characteristic** | **AD (n=21)**  mean (SD)  percentage (counts) | **HC (n=20)**  mean (SD)  percentage (counts) | **HVA (n=5)**  mean (SD)  percentage (counts) |
| --- | --- | --- | --- |
| Age (years) | 38 (±16.5) | 35 (±10.3) | 39 (±12.4) |
| female | 62% (13/21) | 55% (11/20) | 60% (3/5) |
| male | 38% (8/21) | 45% (9/20) | 40% (2/5) |
| EASI | 16.8 (± 12.2) |  | |
| EASI range | 3.6 – 42.4 |  |  |
| age at AD onset | 10.2 (± 12) |  |  |
| allergic rhinitis | 67% (14/21) |  |  |
| allergic asthma | 48% (10/21) |  |  |
| urticaria | 10% (2/21) |  |  |
| eosinophilic esophagitis | 0% (0/20) |  |  |
| sensitization pollen (birch, ragweed) | 52% (11/21) |  |  |
| sensitization dust mite | 67% (14/21) |  |  |
| sensitization food | 14% (3/21) |  |  |
| topical corticosteroid | 67% (14/21) |  |  |

Abbreviations: AD – atopic dermatitis, HC – healthy controls, HVA – hymenoptera venom allergy

**STAT3, STAT5 and STAT6 activation**

Measurement of STAT activation was previously established in the Department of Clinical Immunology at the University of Leipzig^3-5^. Briefly, for analysis of pSTAT3, pSTAT5 and pSTAT6, leucocytes were washed twice and permeabilized (Perm Buffer III, BD Bioscience) for 30 min on ice. After washing, antibody staining was performed using CD3 PerCP-Cy5.5, CD4 PE, CD8 Alexa Fluor 488, Alexa Fluor 647 Mouse Anti-Stat3 (pY705) (all BD Bioscience), Alexa Fluor 647 Mouse Anti-Stat5 (pY694) and Alexa Fluor 647 Mouse Anti-Stat6 (pY641). Gating strategies are shown in Fig S1A.

**Cytokine stimulation**

PMBCs were incubated either with RPMI (baseline) or stimulated (1) for pSTAT3 with human recombinant IL-6 (BD Bioscience, 100 ng/ml); (2) for STAT5 with human recombinant IL-2 (BD Bioscience, 100 ng/ml) and (3) for STAT6 with human recombinant IL-4 (BD Bioscience, 100 ng/ml) for 15 min at 37°C. Additional cytokine stimulation for pSTAT5 included IL-4 (BD Bioscience, 100 ng/ml) and IL-13 (BD Bioscience, 200 ng/ml). PBMCs from 4 healthy donors were isolated, washed with PBS (500xg, 5min RT) and incubated with RPMI (baseline), with RPMI + IL-4 or RPMI + IL-13 for 24h at 37°C, 5% CO2. PBMCs were washed and stained for CD3, CD4, CD8, pStat5 (pY694) (see above).

**Follow up visit**

Four patients receiving different systemic treatments (patient 1: dupilumab 300mg s.c. q2w with loading dose 600mg, patient 2: tralokinumab 300mg s.c. q2w with loading dose 600mg, patient 3: upadacitinib p.o. 30mg once daily and patient 4: systemic methylprednisolone 80mg p.o. (1mg/kg body weight) for 3 days) agreed to participate in a follow up blood draw 4 weeks (patient 4) or 4-6 months (patient 1-3) after initial visit. Patients were advised to treat with topical steroids (starting with mometasone furoate ointment, then tapering with prednicarbate ointment). All patients showed absence of side effects and patients 1-3 continued their treatment after the follow-up visits.

**Immunohistochemistry staining**

Lesional AD skin was taken during diagnostic procedures after informed consent. Control samples were obtained from age-matched individuals undergoing plastic reconstruction after R0 excisions of skin tumours. After providing informed consent, patients donated their Burow’s triangles (No. O93/18-ek). For detection of pSTAT5 (Tyr694) in the skin, FFPE sections were heated at 65 °C for 30 min, deparaffinized, rehydrated, and heated at 100°C for 20 minutes in antigen retrieval buffer (pH 9). Slides were washed, treated with 3% hydrogen peroxide in PBS for 5 minutes, blocked, and incubated with anti-Phospho-Stat5 (Tyr694) (Cell Signaling, C11C5 Rabbit mAb #9359, 1:100 dilution) or anti-Phospho-Stat6 (Y641) (Abcam, ab263947) overnight at 4°C. All slides were then incubated with for 30 minutes with ImmPRESS HRP horse anti-rabbit IgG polymer reagent, followed by incubation with ImmPACT DAB EqV working solution for 10 minutes (ImmPRESS® HRP Horse Anti-Rabbit IgG PLUS Polymer Kit, Vector laboratories, MP-7801), counterstained with hematoxylin, dehydrated, and mounted. Images were acquired using an Olympus BX41TF microscope at indicated magnifications.

**Single cell sequencing analysis and STAT5/STAT6 signature**

We used a recently published single-cell dataset for analysis of T-cell subsets in AD skin^6^. For the STAT5 positive score, we used a gene list consisting of 31 target genes that overlapped in two ChIP sequencing datasets of human T cells^7,8^ (Supplemental Table 1) and applied this gene list to T cell subsets in our scRNA seq dataset. Our STAT6 positive gene score consisted of 32 target genes that were identified in ChIP seq analysis (Supplemental Table 2)^9^. R package Seurat was utilized to apply the module score of genes associated with STAT5A and STAT6 positive expression. The module scores were plotted using R package ggplot2. P-values were calculated between samples using function stat compare means using default setting with pairwise comparisons (R package ggpubr).

**Supplemental Table 1: STAT5A positive signature:**

Target genes positively regulated by STAT5 based on two independent ChIP sequencing datasets.^7,8^

| *ADA* | *EGR3* | *IL2RA* | *PCX* |
| --- | --- | --- | --- |
| *ATF3* | *ETS2* | *IL4* | *SELL* |
| *BCL2* | *EZH2* | *JUN* | *SHE* |
| *CCR4* | *FOS* | *JUNB* | *TNFRSF10B* |
| *CDK6* | *GATA1* | *LDLR* | *TNFRSF9* |
| *CISH* | *GATA2* | *MYO6* | *UBE2C* |
| *CSF2* | *GZMB* | *NR4A1* | *UHRF1* |
| *CXCL10* | *ICOSL* | *NR4A3* |  |
| *CYP11A1* | *IL13* | *NT5E* |  |

**Supplemental Table 2: STAT6 positive signature**

Target genes positively regulated by STAT6 based on ChIP sequencing ^9^

| *IL4* | *AMPH* | *GAB2* | *BCAR3* |
| --- | --- | --- | --- |
| *GATA3* | *TSPAN13* | *C9ORF135* | *ABHD6* |
| *IL24* | *RNF125* | *LOC147645* | *RRS1* |
| *PLCD1* | *MAOA* | *KRT1* | *PTPN14* |
| *HIPK2* | *GPR183* | *CLDN1* | *MYOF* |
| *CRTH2* | *PPP1R14A* | *PLA2G4A* |  |
| *LTB* | *PPARG* | *RUNX1* |  |
| *SOCS1* | *S100P* | *EOMES* |  |
| *CTNS* | *LIMA1* | *ITPRIPL2* |  |

**Statistical analysis**

Statistical analysis was performed using GraphPad Prism version 10.1.0. For comparison of two groups a two-tailed Student’s t-test (normally distributed values) or a Mann‒Whitney U-test (not normally distributed values) was used. Matched values for STAT activation at baseline and after cytokine stimulation with more than two groups were analyzed using 2way ANOVA followed by Šídák's multiple comparisons test. Correlation analysis was performed using Pearson correlation coefficient with EASI values at baseline. Significance was determined by a p value of < 0.05, and annotated as *P < 0.05, **P < 0.01, ***P < 0.001 and ****P<0.0001.

**References**

1 Hanifin, J. M. & Rajka, G. Diagnostic Features of Atopic Dermatitis. *Acta Dermato-Venereologica* **60**, 44-47 (1980). <https://doi.org:10.2340/00015555924447>

2 Hanifin, J. M. *et al.* in *Dermatitis : contact, atopic, occupational, drug* Vol. 33 187-192 (NLM (Medline), 2022).

3 Bitar, M. *et al.* Flow cytometric measurement of STAT5 phosphorylation in cytomegalovirus-stimulated T cells. *Cytometry Part A* **99**, 774-783 (2021). <https://doi.org:https://doi.org/10.1002/cyto.a.24286>

4 Bitar, M. *et al.* Flow cytometric measurement of STAT1 and STAT3 phosphorylation in CD4+ and CD8+ T cells—clinical applications in primary immunodeficiency diagnostics. *Journal of Allergy and Clinical Immunology* **140**, 1439-1441.e1439 (2017). <https://doi.org:https://doi.org/10.1016/j.jaci.2017.05.017>

5 Bitar, M. *et al.* Evaluating STAT5 phosphorylation as a mean to assess T cell proliferation. *Frontiers in Immunology* **10** (2019). <https://doi.org:10.3389/fimmu.2019.00722>

6 Reynolds, G. *et al.* Developmental cell programs are co-opted in inflammatory skin disease. *Science (New York, N.Y.)* **371**, eaba6500 (2021). <https://doi.org:doi:10.1126/science.aba6500>

7 Ding, Z.-C. *et al.* Persistent STAT5 activation reprograms the epigenetic landscape in CD4<sup>+</sup> T cells to drive polyfunctionality and antitumor immunity. *Science Immunology* **5**, eaba5962-eaba5962 (2020). <https://doi.org:10.1126/sciimmunol.aba5962>

8 Villarino, A. *et al.* Signal transducer and activator of transcription 5 (STAT5) paralog dose governs T cell effector and regulatory functions. *eLife* **5**, e08384-e08384 (2016). <https://doi.org:10.7554/eLife.08384>

9 Elo, L. L. *et al.* Genome-wide Profiling of Interleukin-4 and STAT6 Transcription Factor Regulation of Human Th2 Cell Programming. *Immunity* **32**, 852-862 (2010). <https://doi.org:https://doi.org/10.1016/j.immuni.2010.06.011>

**Figure S1.**


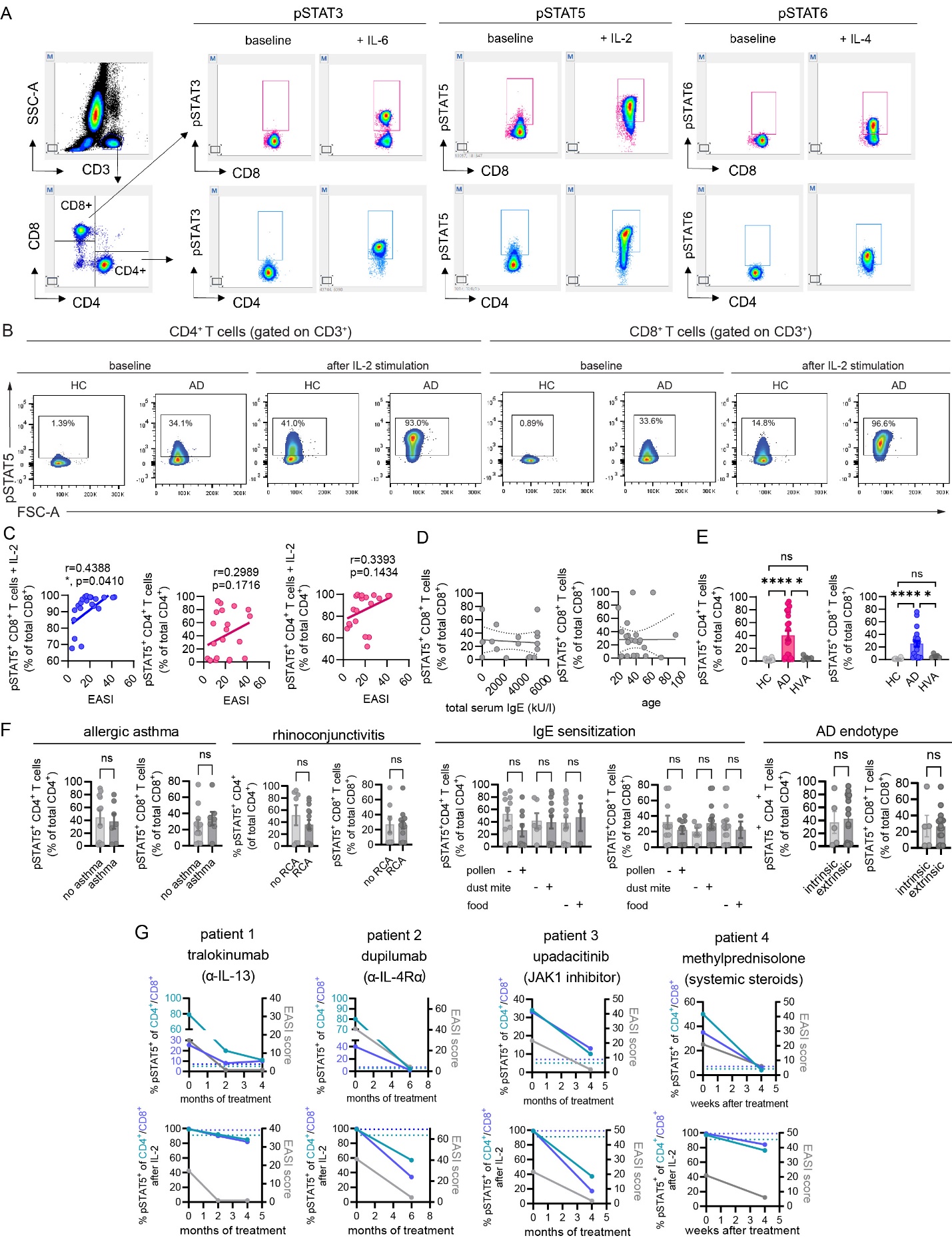


**Figure S1. pSTAT5 activation in AD T cells is independent of atopic comorbidities and sensitizations.**
**A.** Gating strategy. **B.** Example of pSTAT5 activation. **C-D.** Correlation of pSTAT5^+^ T cells after IL-2 with EASI (**C**), with age and total serum IgE (**D**). **E.** pSTAT5^+^ T cells in HC, AD and hymenoptera venom allergy (HVA). **F.** pSTAT5^+^ T cells in patients with allergic asthma, allergic rhinoconjunctivitis (RCA) and with IgE sensitizations to pollen (birch, ragweed), dust mite and food and comparison of pSTAT5^+^ T cell levels in intrinsic (total IgE <114kU/l) and extrinsic (total IgE >114kU/l) AD. **G.** Response of pSTAT5^+^ T cells during therapy in four patients treated with tralokinumab, dupilumab, upadacitinib or short-term systemic steroids, respectively. Two-way ANOVA followed by Šídák's multiple comparisons test, mean and SEM, *P < 0.05, ****P < 0.0001.

**Figure S2.**


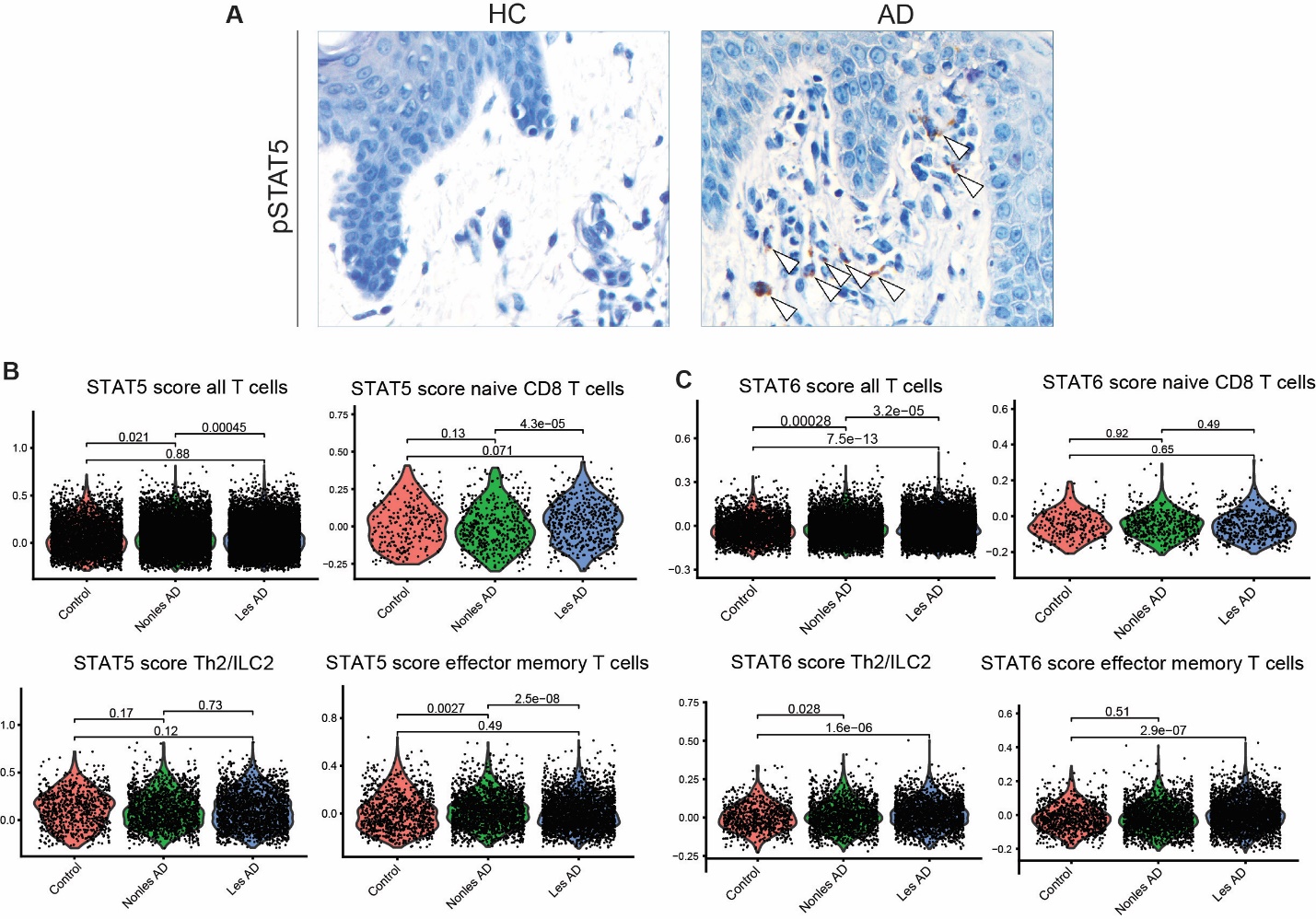


**Figure S2. Module scores reveal STAT5-and STAT6-enriched T cell subsets in AD.**

**A.** Immunohistochemistry of pSTAT5 in HC and AD lesional skin revealed minimal staining in AD. Arrows indicate pSTAT5 positive cells in dermal infiltrate. **B-C.** Violin plots for module scores of STAT5 (**C**) and STAT6 (**C**) in controls, nonlesional AD (nonles) and lesional (les) AD skin samples.
